# Assessment of Depression and Anxiety in Hemodialysis Patients Undergoing VR Therapy—Pilot Study

**DOI:** 10.3390/jcm15041489

**Published:** 2026-02-13

**Authors:** Łukasz Rogowski, Mariusz Kusztal, Joanna Kowalska, Małgorzata Stefańska, Agnieszka Zembroń-Łacny, Tomasz Gołębiowski, Wioletta Dziubek

**Affiliations:** 1 Faculty of Health and Physical Culture Sciences, Witelon Collegium State University, 59-220 Legnica, Poland; 2 Department of Nephrology and Transplantation Medicine, Wroclaw Medical University, 50-367 Wroclaw, Poland; mariusz.kusztal@umw.edu.pl (M.K.); tomasz.golebiowski@umw.edu.pl (T.G.); 3 Faculty of Physiotherapy, Wroclaw University of Health and Sport Sciences, 51-612 Wroclaw, Poland; joanna.kowalska@awf.wroc.pl (J.K.); malgorzata.stefanska@awf.wroc.pl (M.S.); wioletta.dziubek@awf.wroc.pl (W.D.); 4 Department of Applied and Clinical Physiology, Collegium Medicum, University of Zielona Gora, 65-417 Zielona Gora, Poland; a.zembron-lacny@inz.uz.zgora.pl

**Keywords:** hemodialysis, end-stage renal disease, VR therapy, depression, anxiety, self-efficacy, stress, life satisfaction

## Abstract

**Background/Objectives:** Patients with end-stage renal disease (ESRD) are at a significant risk for depressive and anxiety disorders, including severe depression. The use of virtual reality (VR) during hemodialysis can significantly impact psychological well-being and thus overall quality of life. The aim of the study was to assess the effects of VR therapy undertaken by hemodialysis (HD) patients on depression, anxiety, perceived self-efficacy and life satisfaction. **Methods:** Hemodialysis (HD) patients were recruited from the Dialysis Centre at St. Luke’s Hospital in Bolesławiec. Group I (experimental EG) included 16 HD patients, and Group II (control CG) included 22 HD patients (no VR session). The EG used VR for one month (maximum 14 sessions); the CG was the same group of patients assessed over a one-month period prior to the intervention. VR therapy took place at the beginning of dialysis, three times a week for one month. A personal questionnaire, the Hospital Anxiety and Depression Scale (HADS), the Perceived Stress Scale (PSS-10), the General Self-Efficacy Scale (GSES) and the Satisfaction with Life Scale (SWLS) were used. **Results:** In the group of patients undergoing VR therapy, a significant reduction in the level of anxiety, depression and stress was observed, as well as a significant increase in life satisfaction and self-efficacy levels compared to the control group. **Conclusions**: Undertaking VR therapy during dialysis for patients with ESRD is beneficial, improves mood, satisfaction with life, and perceived self-efficacy, and reduces anxiety, but multi-center studies are needed on a larger group of patients.

## 1. Introduction

Patients with end-stage renal disease (ESRD) are at significant risk of depressive and anxiety disorders, including severe depression. Depression affects approximately 30% of the population in this group of patients [1]. These disorders not only reduce the quality of life of patients but also increase the risk of mortality (up to 50%), complications of ESRD, hospitalization, and substance use [2,3]. The presence of depression not only worsens the treatment process, including the hemodialysis process itself by reducing adherence to dialysis, but also worsens the patient’s prognosis after kidney transplantation [4].

The development of depressive and anxiety disorders may be driven by specific, disease-related biochemical changes [5,6], as well as nonspecific, difficult-to-define behavioral variables resulting from the patient’s relationship with the disease, treatment prospects, and life situation. End-stage renal disease leads to multiple, sudden, surprising, and often serious health crises [4].

The literature also emphasizes the psychological aspect—the patient’s relationship with the dialysis treatment itself: anxiety related to the invasive procedures associated with hemodialysis, as well as a certain dependence on the machine (hemodialyzer) [7]. The problem of these disorders and their significant seriousness has long been recognized. Primary psychiatric treatment is, of course, used in the form of pharmacological therapy, but it is important to remember the significant burden of primary treatment for these patients, both for the primary illness and secondary conditions (polypharmacy), and the increased risk of side effects of antidepressant treatment [8]. Therefore, attempts are also being made to introduce alternative solutions in the form of psychotherapeutic methods, but it should be noted that these methods are complex, requiring qualified personnel and significant financial and organizational resources [9].

Patients often perceive dialysis as a time removed from life, with a sense of inevitability and emptiness, triggering negative emotional and cognitive perceptions of the world and their place in it. Fatigue, anxiety, fear, and depression can exacerbate treatment side effects and reduce its effectiveness. Therefore, therapeutic interventions that increase treatment tolerance are crucial for the quality of life of dialysis patients.

Hence, the need has arisen to develop simpler methods with broader applications. Virtual reality (VR) and experience from other medical conditions come to the rescue. The use of virtual reality during hemodialysis can have a significant impact on mood, motivation, perceived self-efficacy, and thus on overall quality of life. The potential of virtual reality (VR) is already being utilized with promising results in the treatment of psychiatric patients. The powerful audio–visual stimulus itself, which engages the patient in the process of receiving and processing information, has the potential to reduce anxiety and depression [10]. Previous literature reviews have assessed the effectiveness of virtual reality-based interventions in the treatment of anxiety and depression symptoms in older adults, cardiac patients, stroke patients, and oncology patients [11,12].

The use of VR can give patients with ESRD a sense of being cared for and receiving modern, innovative therapies, even for such a chronic and challenging condition. Utilizing the phenomenon of immersion in a virtual world can be a way to distract from everyday worries and anxieties.

Due to the frequent occurrence of low mood and high levels of stress and anxiety in dialysis patients, researchers are seeking methods that could improve their emotional well-being while utilizing the time spent on dialysis. Improving mood and reducing anxiety is associated not only with increased motivation but also with improved self-efficacy. This builds self-confidence, provides a sense of regained control, and thus increases patient engagement in the treatment and therapy process, which translates into greater independence and, above all, an adherence to treatment recommendations. The benefits of VR therapy may translate into greater patient engagement in more demanding activities (e.g., strength or endurance training) during dialysis, thus improving quality of life.

The aim of the study was to assess the emotional state (mood, anxiety, stress and perceived self-efficacy) and its changes in a group of dialysis patients undergoing virtual reality (VR) relaxation therapy. Hypotheses: Intradialytic VR relaxation therapy will reduce the symptoms associated with depression and anxiety in hemodialysis patients and improve perceived self-efficacy.

## 2. Materials and Methods

### 2.1. Study Design

Hemodialysis (HD) patients were recruited from the Dialysis Centre at St. Luke’s Hospital in Bolesławiec, Poland. This pilot study presents grant results from the Wroclaw University of Health and Sport Sciences, no. Z.23.03. The study protocol was approved by The Ethics Committee of the Wroclaw University of Health and Sport Sciences, Poland (reference no 9/2024). The study was conducted in accordance with the Helsinki Declaration. Written consent was obtained from the patients for participation in the study, which was preceded by an explanation of the purpose and course of the study, assurance of confidentiality of the collected data, and information about the voluntary nature of their participation and the possibility of withdrawing at any time during the project.

### 2.2. Participants

All participants were recruited for participation in the program by a nephrologist and a psychologist, who administered the Mini-Mental State Examination (MMSE) to assess the cognitive status of patients. Patients had to meet the following inclusion criteria:

- Consent to participate in the study,
- Diagnosed chronic renal failure (CKD),
- Hemodialysis treatment for at least 3 months prior to the start of the study,
- Adequate dialysis therapy: dialysis adequacy index Kt/V > 1.2; protein catabolism coefficient (PCR) 0.8–1.4 g protein/kg body weight/day,
- Ability to perform a full cognitive function test,
- Satisfactory cognitive function (MMSE > 23) enabling reliable psychological testing,
- Age 40–70 years.

Exclusion criteria were:

- Contraindications to VR training (epilepsy, vertigo, labyrinth disorders, motion sickness),
- Presence of serious mental disorders in medical records (e.g., impaired consciousness, dementia),
- Adherence to training units below 70%,
- Medication-treated depressive disorders or other mental disorders,
- Use of other non-pharmacological forms of support during the project,
- Lack of consent to participate in the study.

### 2.3. Design of the Study

**Figure 1:**
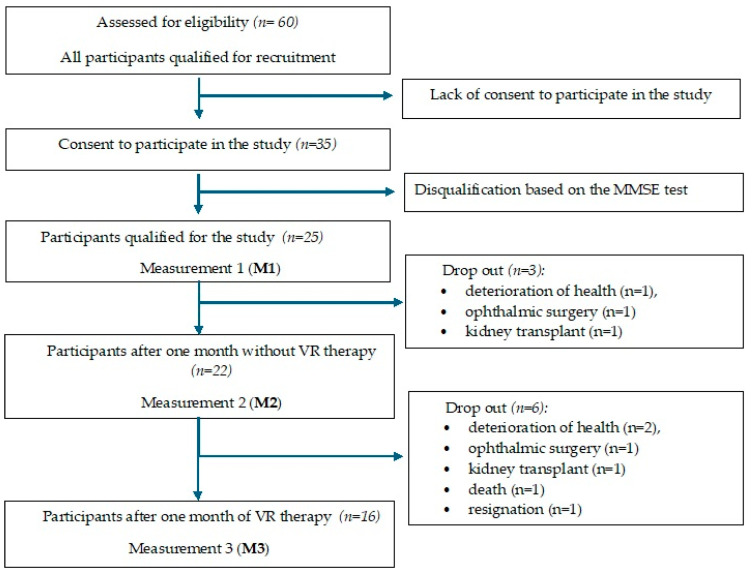
Flow diagram of the study.

All psychometric tests were administered at three time points within the same group of patients: at the start of the study—measurement 1 (M1); after one month without VR therapy—measurement 2 (M2); and after one month with VR therapy—measurement 3 (M3). That is, the experimental group used VR for one month, and the control group was the same group of patients assessed over one month before the intervention (Figure 1). To ensure adherence to the study, the patients were informed about the planned VR intervention. The characteristics of the study group starting VR therapy are presented in Table 1.

Relaxation therapy was conducted using immersive VR goggles on the same group of patients. VR therapy took place at the beginning of dialysis, three times a week for one month (maximum 14 sessions). The relaxation therapy was applied from 20 min, gradually increasing to a maximum of 50 min, and was conducted under the constant supervision of a nephrologist and a physiotherapist. Patients were also informed that VR therapy could be discontinued if adverse reactions occurred (e.g., nausea, dizziness, visual discomfort).

At the beginning of the intervention, a short interview was conducted with each patient to explain and introduce the patient to the technical aspects of using VR goggles and safety rules. During the passive VR therapy, various realistic scenarios were presented, including selected 360° videos and standard 2D landscape videos with relaxation music. Active, full-immersive applications requiring the operation of controllers were excluded for safety reasons. After every session, a short form interview was conducted to assess any adverse reactions. There were no resignations noted due to adverse reactions to VR therapy.

### 2.4. Measurement Tools

A personal questionnaire was used to describe the characteristics of the group.

Additionally, the following scales were used: the Hospital Anxiety and Depression Scale (HADS), the Perceived Stress Scale (PSS-10), the General Self-Efficacy Scale (GSES), and the Satisfaction with Life Scale (SWLS).

#### 2.4.1. HADS

The Hospital Anxiety and Depression Scale (HADS) is a screening tool used to assess the severity of anxiety and depression symptoms, particularly in patients treated in hospitals and outpatient clinics. The scale consists of 14 questions, divided into two subscales: HADS-A assesses anxiety-related symptoms, and HADS-D measures depressed mood, lack of joy, decreased energy, and anhedonia [13].

#### 2.4.2. PSS-10

The Perceived Stress Scale assesses the intensity of perceived stress. It contains 10 questions about various subjective feelings related to personal problems and events, behaviors, and coping methods. The overall score is the sum of all points, ranging from 0 to 40. The higher the score, the greater the intensity of perceived stress [14].

#### 2.4.3. GSES

The General Self-Efficacy Scale is a tool containing 10 statements that comprise a single factor. It measures the strength of an individual’s overall belief in the effectiveness of coping with difficult situations and obstacles. It is designed for adults, both healthy and sick. Self-efficacy allows for the prediction of intentions and actions in various areas of human activity, including health-related behaviors [15,16].

#### 2.4.4. SWLS

The Satisfaction with Life Scale measures life satisfaction. This instrument consists of five statements, which the respondent rates on a seven-point scale. Respondents assess the extent to which each statement applies to their life to date. Life satisfaction assessed on the SWLS expresses a sense of satisfaction with one’s achievements and life situation [17].

### 2.5. Statistical Analysis

A normal distribution of quantitative variables describing the study group was not demonstrated. Descriptive statistics were calculated. Due to the ordinal nature of most variables and the lack of normal distribution of quantitative variables, the median was used as a measure of central tendency, and the interquartile range (IQR = Q3–Q1) was used as a measure of dispersion. The significance of differences between the three repeated measurements was tested using Friedman ANOVA. If the observed differences were significant, the Dunn–Bonferroni–Holm post hoc test was used to indicate which measurements had a significant difference. Effect size was tested using Kandall’s W coefficient. The analysis was performed using Statistica 14.1.0.4 and PQStat 1.8.4. The level of significance was set at *p* < 0.05.

## 3. Results

### 3.1. HADS

The assessment of anxiety and depression levels conducted using the HADS questionnaire has shown a significant reduction in anxiety symptoms (HADS-A), depressive symptoms (HADS-D), and general scores (HADS total) after the period of VR relaxation treatment—between M2 and M3. In the control period, between the results of M1 and M2, no significant differences were observed (Table 2, Table 3 and Table 4).

### 3.2. PSS-10

The level of perceived stress, estimated using the PSS-10 questionnaire, was moderate at M1 and M2 and low at M3. The analysis of the significance of differences between measurements showed no differences between M1 and M2. A significant reduction in perceived stress symptoms was observed between M1 and M3 and between M2 and M3, that is, after the VR therapy period (Table 5).

### 3.3. SWLS

The analysis of the obtained results showed a significant increase in life satisfaction assessed after one month of VR therapy (M3), compared to the control period (M1 and M2). At M1 and M2, the level of life satisfaction of HD patients could be described as moderately high, while at M3 it was high (Table 6).

### 3.4. GSES

The GSES results indicated high levels of patient self-efficacy at each of the three measurement points. Statistically significant differences were found only between M2 and M3, that is, the VR therapy period (Table 7).

## 4. Discussion

Chronically ill patients require long-term treatment and are at risk of long-term side effects, treatment complications, and rehospitalizations. The background for the development of depressive and anxiety disorders can be disease-specific and related to biochemical changes [5,6], as well as non-specific, difficult-to-define behavioral variables. A source of anxiety for long-term HD patients is undoubtedly their current health status and the awareness of reduced control over their own health. Hemodialysis patients may experience severe stress, not only due to the primary disease, but also due to possible adverse events during dialysis [18]. This exposes them to increased stress and mood disorders [19]. These disorders entail not only a reduced quality of life for patients but also an increased risk of mortality (by up to 50%), complications of renal disease, hospitalization, and use of addictive substances. The presence of depression not only worsens the treatment process of patients but also worsens the prognosis of the patient after kidney transplantation [1].

The literature also emphasizes the psychological aspect—the attitude to dialysis treatment itself: anxiety associated with the invasive procedures accompanying hemodialysis and potential complications, as well as a certain dependence on the machine (hemodialyzer) [20].

Various psycho-physiological treatments were of clinical interest for a long time. Muscle relaxation techniques, breathing exercises, or mindfulness meditations can significantly stimulate the parasympathetic system, thus causing a reduction in stress and mood disorders [21].

VR therapies are successfully applied in many psychiatric and somatic disorders. The significant positive results are observed for, e.g., anxiety disorders, PTSD or cognitive decline disorders [10]. Primary psychiatric treatment in the form of pharmacological treatment is widely used, but it is important to keep in mind the significant burden of the primary treatment of these patients. Multiple pharmacological treatments increase the risk of side effects [8]. Therefore, attempts are also being made to introduce alternatives in the form of psychotherapeutic methods. However, it should be taken into account that these methods are complex, requiring qualified personnel and significant financial and organizational resources [8,22,23].

Hence, there was a need to develop simpler methods with wider application. The immersive VR separates the user from the real environment and allows the user to immerse themselves in a three-dimensional computer-generated environment. The user can passively observe the presented virtual reality or even interact with it. Thus, immersion through VR can result in a stronger emotional influence on the user and a greater physiological response. Another beneficial factor of immersive VR is the possibility of applications in special conditions with limited space or personnel.

There are still insufficient reports regarding the influence of VR therapies on HD patients. All the up-to-date reported VR trials include physical exercise, although these experiments have also measured outcomes related to depressive symptoms and quality of life. In general, implemented VR treatments have shown a small but significant beneficial effect in HD patients. Although there was no particular VR relaxation therapy analyzed, exercise is a strong confounding factor [24].

The meta-analysis by Kang et al. [25] also indicates positive results of such interventions, citing only four studies examining the impact on mood disorders and anxiety. Unfortunately, some publications are unavailable. The cited studies also involved the use of active physical exercises in a VR environment to improve functional status. Gurz et al., in their narrative review, again mainly collected studies focusing on the introduction of physical exercises in a VR environment, citing only one study with a methodology and assumptions similar to ours [26].

Our aim, however, was to examine the impact of VR therapy using the least invasive and safest method possible, without the need for patient activation, specialized methods, or specialists. By introducing simple, safe relaxation in a VR environment, we observed significant reductions in depression symptoms and anxiety and stress levels. This may be explained by a number of mechanisms. It is important to note that dialysis is a lengthy, tedious experience, taking place in a hospital setting, which can lead to anxiety disorders [20].

The observed reductions in perceived stress, anxiety, and depressive disorders may result from short-term mechanisms of distraction from the surroundings and concentration on the content presented in the VR environment. It can also be assumed that, in the long term, the awareness of the enrichment of the hemodialysis procedure itself with a simple procedure may impact the patient’s well-being and anticipation of dialysis.

The literature on the subject points out that relaxation methods, especially in an immersive VR environment, can effectively reduce the intensity of stress and pain associated with medical procedures [27]. Hsieh et al. used a series of short 360-degree videos to observe the beneficial effects of the intervention on sympathetic nervous system function and stress reduction, as expressed by the heart rate during dialysis (HR and HRV), reaching similar conclusions [28].

In CVD patients, implemented VR therapy resulted in a reduction in anxiety and depressive symptoms [29]. The beneficial impact of VR relaxation on anxiety and depressive symptoms was also observed in pulmonary patients [12].

However, Smyth et al. did not observe a significant effect of VR therapy on patients’ mental well-being [30]. However, the explanation may lie in the graphical differences between the videos presented in the VR environment. Smyth et al. used short 360-degree videos generated in a graphics program, which are not actually realistic videos of the surroundings. In our study, we used realistic images of nature presented in a 360-degree environment or in a classic 2D environment, which may enhance perception and presence, increase immersion, and more effectively influence the attention and mood of patients [30]. The passive relaxation we have used is much more accessible and safer for patients, and does not require active supervision by medical personnel, increasing the application value of the intervention.

VR therapy improved self-efficacy and life satisfaction in the dialysis patients studied. This is significant because it promotes improved motivation, builds self-confidence, and thus increases patient engagement in the treatment and therapy process, which translates into a greater independence and adherence to treatment recommendations [25,31,32].

The above results clearly demonstrate that a low self-efficacy is associated with depression, anxiety, and helplessness, while improved emotional well-being translates into increased self-efficacy. Therefore, an individual’s confidence in their own abilities to cope with various situations, including illness, may be crucial in the treatment and rehabilitation process.

### Limitations

The main limitation of this study is the small size of the group that participated in the training. The tests used are screening in nature and do not constitute a diagnosis. The study was a pilot study conducted at a single center. Furthermore, the intervention lasted one month, without an extended follow-up period, so the durability of the effects cannot be determined.

## 5. Conclusions

The obtained research results are promising and indicate the benefits of using VR therapy during dialysis for patients with ESRD. In the study group, VR therapy improves mood, life satisfaction, and self-efficacy levels and reduces anxiety, but multi-center studies are needed for a larger group of patients.

## Figures and Tables

**Table 1:** Characteristics of the study group (*n* = 22).

		Median	IQR
Age [years]		69.00	23.00
Height [m]		1.72	0.12
Body weight [kg]		85.85	31.00
BMI [kg/m^2^]		26.75	10.20
Years of dialysis		3.00	2.00
		*n*	%
Gender	M	12	52.17
	W	10	43.48
Place of residence	Rural area	12	52.17
	Small town	9	39.13
	Big city	1	4.35
Marital status	Married (M)	11	47.83
	Married (W)	5	21.74
	Widowed	3	13.04
	Single	2	8.70
	Divorced	1	4.35
Education	Vocational	5	21.74
	Secondary	10	43.48
	Elementary	4	17.39
	Higher	3	13.04

IQR—interquartile range, M—men, W—women.

**Table 2:** Significance of differences between Hospital Anxiety and Depression Scale—Anxiety (HADS-A) scores.

				Friedman ANOVA	Post Hoc (Dunn–Bonferroni–Holm)			Kendall’s W
	Timeline	Median	IQR	*p*	1 vs. 2	1 vs. 3	2 vs. 3	
HADS-A	M1	7	4	<0.0001	0.7237	0.0003	0.0001	0.75
	M2	7.5	5					
	M3	4	5					

IQR—interquartile range; HADS-A—Hospital Anxiety and Depression Scale (Anxiety); M1—measurement 1; M2—measurement 2; M3—measurement 3; *p* < 0.05.

**Table 3:** Significance of differences between Hospital Anxiety and Depression Scale—Depression (HADS-D) scores.

				Friedman ANOVA	Post Hoc (Dunn–Bonferroni–Holm)			Kendall’s W
	Timeline	Median	IQR	*p*	1 vs. 2	1 vs. 3	2 vs. 3	
HADS-D	M1	6	7	0.0002	0.1116	0.0340	0.0002	0.54
	M2	9	3					
	M3	3	4.5					

IQR—interquartile range; HADS-D—Hospital Anxiety and Depression Scale (Depression); M1—measurement 1; M2—measurement 2; M3—measurement 3; *p* < 0.05.

**Table 4:** Significance of differences between Hospital Anxiety and Depression Scale scores (total).

				Friedman ANOVA	Post Hoc (Dunn–Bonferroni–Holm)			Kendall’s W
	Timeline	Median	IQR	*p*	1 vs. 2	1 vs. 3	2 vs. 3	
HADS (total)	M1	15	10	<0.0001	0.2888	0.0011	<0.0001	0.73
	M2	17	7					
	M3	6.5	8.5					

IQR—interquartile range; HADS (total)—Hospital Anxiety and Depression Scale; M1—measurement 1; M2—measurement 2; M3—measurement 3; *p* < 0.05.

**Table 5:** Significance of differences between the Perceived Stress Scale (PSS-10) scores recorded at three measurement points.

				Friedman ANOVA	Post Hoc (Dunn–Bonferroni–Holm)			Kendall’s W
	Timeline	Median	IQR	*p*	1 vs. 2	1 vs. 3	2 vs. 3	
PSS-10	M1	19	12	<0.0001	1.0000	0.0001	0.0001	0.77
	M2	18.5	12					
	M3	11.5	9					

IQR—interquartile range; PSS-10—Perceived Stress Scale; M1—measurement 1; M2—measurement 2; M3—measurement 3; *p* < 0.05.

**Table 6:** Significance of differences between Satisfaction with Life Scale (SWLS) scores recorded at three measurement points.

				Friedman ANOVA	Post Hoc (Dunn–Bonferroni–Holm)			Kendall’s W
	Timeline	Median	IQR	*p*	1 vs. 2	1 vs. 3	2 vs. 3	
SWLS	M1	22.5	9	<0.0001	0.8597	0.0001	<0.0001	0.79
	M2	21	9					
	M3	28	5.5					

IQR—interquartile range; SWLS—Satisfaction with Life Scale; M1—measurement 1; M2—measurement 2; M3—measurement 3; *p* < 0.05.

**Table 7:** Significance of differences between the Generalized Self-Efficacy Scale (GSES) scores recorded at three measurement points.

				Friedman ANOVA	Post Hoc (Dunn–Bonferroni–Holm)			Kendall’s W
	Timeline	Median	IQR	*p*	1 vs. 2	1 vs. 3	2 vs. 3	
GSES	M1	35.5	6	0.0393	0.2854	0.3159	0.0407	0.15
	M2	32	10					
	M3	35	13					

IQR—interquartile range; GSES—General Self-Efficacy Scale; M1—measurement 1; M2—measurement 2; M3—measurement 3; *p* < 0.05.

## Data Availability

The data presented in this study are available from the corresponding author upon request.

## Abbreviations

The following abbreviations are used in this manuscript:

HD	Hemodialysis
ESRD	End-Stage Renal Disease
VR	Virtual Reality
EG	Experimental Group
CG	Control Group
MMSE	Mini-Mental State Examination
HADS	Hospital Anxiety and Depression Scale
PSS-10	Perceived Stress Scale
GSES	General Self-Efficacy Scale
SWLS	Satisfaction with Life Scale
